# Metabolic versatility of anaerobic sludge towards platform chemical production from waste glycerol

**DOI:** 10.1007/s00253-024-13248-6

**Published:** 2024-07-16

**Authors:** Carla P. Magalhães, Joana I. Alves, Anna Duber, Piotr Oleskowicz-Popiel, Alfons J. M. Stams, Ana J. Cavaleiro

**Affiliations:** 1 CEB - Centre of Biological Engineering, University of Minho, Braga, Portugal; 2 LABBELS - Associate Laboratory, Braga/Guimarães, Portugal; 3 Water Supply and Bioeconomy Division, Faculty of Environmental Engineering and Energy, Poznan University of Technology, Poznan, Poland; 4 Laboratory of Microbiology, Wageningen University & Research, Wageningen, The Netherlands

**Keywords:** Glycerol, Methane, Acetate, Propionate, Microbial communities, Metabolism

## Abstract

**Abstract:**

Waste glycerol is produced in excess by several industries, such as during biodiesel production. In this work, the metabolic versatility of anaerobic sludge was explored towards waste glycerol valorization. By applying different environmental (methanogenic and sulfate-reducing) conditions, three distinct microbial cultures were obtained from the same inoculum (anaerobic granular sludge), with high microbial specialization, within three different phyla (*Thermodesulfobacteriota*, *Euryarchaeota* and *Pseudomonadota*). The cultures are capable of glycerol conversion through different pathways: (i) glycerol conversion to methane by a bacterium closely related to *Solidesulfovibrio alcoholivorans* (99.8% 16S rRNA gene identity), in syntrophic relationship with *Methanofollis liminatans* (98.8% identity), (ii) fermentation to propionate by *Propionivibrio pelophilus* strain asp66 (98.6% identity), with a propionate yield of 0.88 mmol mmol^−1^ (0.71 mg mg^−1^) and a propionate purity of 80–97% and (iii) acetate production coupled to sulfate reduction by *Desulfolutivibrio sulfoxidireducens* (98.3% identity). In conclusion, starting from the same inoculum, we could drive the metabolic and functional potential of the microbiota towards the formation of several valuable products that can be used in industrial applications or as energy carriers.

**Key points:**

- *Versatility of anaerobic cultures was explored for waste glycerol valorization*
- *Different environmental conditions lead to metabolic specialization*
- *Biocommodities such as propionate, acetate and methane were produced*

**Supplementary Information:**

The online version contains supplementary material available at 10.1007/s00253-024-13248-6.

## Introduction

Biodiesel production and ethanol production by yeast or the oleochemical industry generate glycerol as a by-product (Clomburg and Gonzalez 2013; Monteiro et al. 2018; Navarrete et al. 2014). Largely exceeding its demand, glycerol changed from a commodity chemical to a surplus by-product and even to a waste product, creating environmental and economic losses (Clomburg and Gonzalez 2013; Monteiro et al. 2018; Viana et al. 2012). Within this framework, anaerobic bioconversion of glycerol to valuable chemical compounds can be a sustainable treatment strategy, adding value to waste glycerol and to the biodiesel industry (Holm-Nielsen et al. 2009; Viana et al. 2012; Yazdani and Gonzalez 2007).

Under anaerobic conditions, the high reduction state of glycerol is an advantage, as glycerol fermentation results in the production of more reduced compounds than with sugars as glucose (Yazdani and Gonzalez 2007). Nevertheless, the high reduction state of glycerol also presents considerable challenges, since only a few fermentative bacteria are capable of easily disposing off the excess of reducing equivalents generated from glycerol. Other bacteria can oxidize glycerol coupled to the reduction of external electron acceptors, such as sulfate (Clomburg and Gonzalez 2013), or in syntrophy with hydrogenotrophic methanogens (Qatibi et al. 1991a, b). Syntrophic collaboration was even shown to accelerate glycerol degradation (Magalhães et al. 2020; Richter and Gescher 2014), possibly because it facilitates the maintenance of the proper intracellular redox balance. Zhang et al. (2015) suggested that the use of mixed cultures for glycerol degradation may present economic and process advantages.

The objective of this work was to drive the naturally occurring microbiome of anaerobic sludge towards glycerol consumption and valorization and to study the diversity and physiology of the obtained microorganisms and/or communities. Starting from the same inoculum (anaerobic sludge), three distinct specialized glycerol degrading cultures were obtained, and their physiology was studied. The obtained cultures are capable of metabolizing glycerol through different pathways, proving the metabolic versatility of using anaerobic mixed cultures, as well as proving the concept of the ability to shape mixed microbial communities towards specific needs (Oleskowicz-Popiel 2018).

## Materials and methods

### Enrichment of glycerol-degrading cultures

Enrichments were made in 120 mL serum bottles containing 50 mL of a bicarbonate-buffered mineral salt medium (basal medium, BM), prepared as previously described by Stams et al. (1993). The serum bottles were sealed with butyl rubber septa and aluminum crimp caps, and the headspace of the bottles was flushed and pressurized with N_2_/CO_2_ (80:20%, v/v) at a final pressure of 170 kPa. The medium was reduced with 1 mmol L^−1^ sodium sulfide and supplemented with salts and vitamins (Stams et al. 1993). Anaerobic granular sludge from a brewery wastewater treatment plant (Portugal) was used as inoculum. Glycerol (10 mmol L^−1^) was supplemented as a carbon and energy source. Enrichments were developed in the absence of any added external electron acceptor (methanogenic conditions), resulting in two different cultures coded as Gly-M and Gly-P or with 20 mmol L^−1^ sodium sulfate (sulfate-reducing conditions), coded as Gly-S. Successive transfers (10% v/v) were made to fresh medium after confirming glycerol consumption and microbial growth in all assays. Methane content in the bottles’ headspace and the concentration of soluble compounds, such as volatile fatty acids (VFA), lactate, aspartate, succinate, glycerol, ethanol, butanol, 1,3-propanediol (1,3-PDO) and 1,2-propanediol (1,2-PDO), were periodically measured. In the Gly-S set of experiments, sulfate reduction was assessed indirectly by the amount of sulfide produced (Eq. 1).

1 $${{\mathrm{SO}}_{4}}^{2-}+9\text{ H}^{+}+{8\text{ e}}^{-} {\to \mathrm{HS}}^{-}+4 {\mathrm{H}}_{2}\mathrm{O}$$

**Fig. 1:**
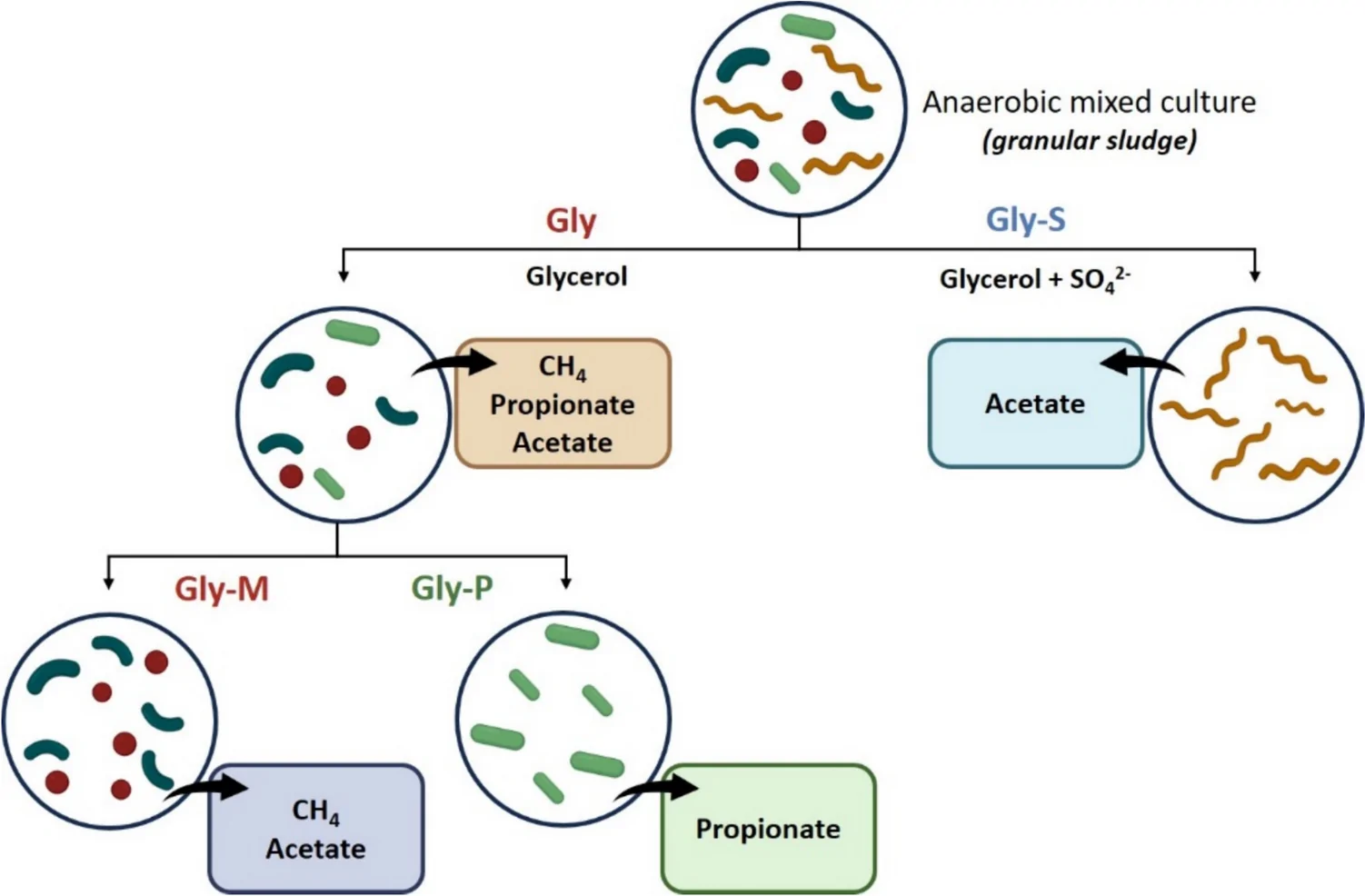
Schematic representation of the enrichments performed; the compounds shown in the boxes are the main products of glycerol conversion

All inoculations and transfers were done aseptically. Incubations were performed at 37 °C, statically and in the dark. A schematic representation of the experiments is shown in Fig. 1 (“Results” section).

### Physiological characterization of Gly-M-, Gly-P- and Gly-S-enriched cultures

Physiological characterization was done with the stable cultures of Gly-M, Gly-P and Gly-S after 15, 8 and 10 successive transfers, respectively. Unless otherwise stated, incubations were performed with 10 mmol L^−1^ glycerol as a carbon source. For Gly-S, sodium sulfate was used as the final electron acceptor at 20 mmol L^−1^. In the case of enrichment culture Gly-M, incubations with 2-bromoethanesulfonate (BrES), a specific inhibitor of the methanogens (20 mmol L^−1^), were also performed.

Purity check was done in Gly-P- and Gly-S-enriched cultures by microscopic examination after incubation with yeast extract (2 g L^−1^), glucose (10 mmol L^−1^), or pyruvate (10 mmol L^−1^). Cells from active cultures of Gly-P and Gly-S were Gram-strained and cell morphology was examined by phase contrast microscopy.

Physiological characterization of these two cultures was performed in the presence of different glycerol concentrations: 10, 30, 50, 100 and 200 mmol L^−1^. Supplemental Table S1 summarizes all the procedures applied.

For Gly-P-enriched culture, the ability of this culture to degrade ethanol, propanol or 1-butanol (10 mmol L^−1^), aspartate (20 mmol L^−1^) or succinate (20 mmol L^−1^) was tested as well. In regard to Gly-S culture, the capability to use ethanol, propanol and 1-butanol at different concentrations (10, 20, 30 and 40 mmol L^−1^) was also investigated. In the case of Gly-S, all incubations were done with (20 mmol L^−1^) or without sulfate as an electron acceptor. Additionally, using the obtained Gly-S culture, potential syntrophic growth with a methanogenic partner, in the absence of sulfate, was assessed. For that, *Methanobacterium formicicum* DSM 1535^ T^ was acquired from the Deutsche Sammlung von Mikroorganismen und Zellkulturen (DSMZ, Braunschweig, Germany). The methanogen was pre-grown with H_2_/CO_2_ (80:20% v/v, at a final pressure of 170 kPa) in BM medium supplemented with 0.3 g L^−1^ sodium acetate, at 37 °C and 100 rpm. After the headspace was changed to N_2_/CO_2_ (80:20% v/v, 170 kPa) under sterile conditions, glycerol was added (10 mmol L^−1^) and well-grown Gly-S culture was transferred (10% v/v) to these bottles. The Gly-S culture was also used to inoculate (10% v/v) control bottles, containing fresh medium, glycerol (10 mmol L^−1^) and sulfate (20 mmol L^−1^). After verifying the growth and activity of the co-cultures (Gly-S + methanogenic partner), these were transferred again (10% v/v) to bottles containing pre-grown cultures of the methanogen (prepared as described before) and glycerol (10 mmol L^−1^).

Substrate consumption, liquid (soluble) and gaseous product formation, sulfate reduction (assessed indirectly by the sulfide produced) and cell growth were monitored over time, for all the experiments, as described in the “Analytical methods” section.

Cultures Gly-P and Gly-S were deposited in culture collections belonging to the World Data Centre for Microorganisms (WDCM), as detailed in the data availability statement. Regarding the enrichment culture Gly-M, it is accessible at the Laboratory of Environmental Technology from the Centre of Biological Engineering of the University of Minho (Braga, Portugal), with A.J. Cavaleiro.

### Microbial communities’ composition

Microbial community composition of stable enriched cultures Gly-M, Gly-P and Gly-S was evaluated through 16S rRNA gene sequencing. Aliquots (15 mL) of well-homogenized stable enrichment cultures were collected from Gly-M, Gly-P and Gly-S, and immediately frozen at − 20 °C. Total genomic DNA was extracted using the FastDNA SPIN Kit for Soil (MP Biomedicals, Solon, OH) and purified by ethanol precipitation. Bacterial and archaeal 16S rRNA genes were amplified using a TaqDNA polymerase kit (Invitrogen, Carlsbad, CA) and the primers Bact27-F7/Uni1492-R and Arch109-F/Uni1492-R, respectively. PCR programs and reaction mixtures used were described elsewhere (Sousa et al. 2007). Cloning and Sanger sequencing of the obtained 16S rRNA genes were performed using the methodologies previously described by Sousa et al. (2007). Sanger sequencing method was performed by Macrogen Europe (Amsterdam, ND), and the obtained sequences were compared with the NCBI RefSeq_RNA database using the NCBI Nucleotide Blast tool (https://www.ncbi.nlm.nih.gov/nucleotide/). Nucleotide sequencing was submitted to the European Nucleotide Archive (ENA) under the study accession no. PRJEB72408.

### Analytical methods

Phase contrast micrographs were obtained with an Olympus CX41 RF microscope (Tokyo, Japan) and an Olympus Altra 20 image acquisition system. The software used with this setup was the AnalySIS getIT (Olympus Soft Imaging Solutions GmbH). The Gram staining was performed as previously described by Halebian et al. (1981). Methane was quantified with a GC-2014 Shimadzu gas chromatograph equipped with a Porapak Q column and a flame ionization detector. N_2_ was used as carrier gas. Injection port, column and detector temperatures were 110 °C, 35 °C and 220 °C, respectively. VFA (formate, acetate, propionate, *iso-* and *n-*butyrate and valerate) and lactate, succinate and aspartate were analyzed by high-performance liquid chromatography (HPLC, Jasco, Tokyo, Japan), using an Agilent Hi-Plex H (300 × 7.7 mm) column at 60 °C and H_2_SO_4_ (2.5 mmol L^−1^) as mobile phase, at a flow rate of 0.6 mL min^−1^. Spectrophotometric ultraviolet (UV) detection was performed at 210 nm. Glycerol, ethanol, butanol, 1,3-PDO and 1,2-PDO were analyzed by HPLC using a Varian Aminex 87H (300 × 7.8 mm) column with a mobile phase of 5 mmol L^−1^ H_2_SO_4_ at a flow rate of 0.7 mL min^−1^, with the column temperature set at 60 °C and refractive index (RI) detection. Total dissolved sulfide was measured using cuvette tests and a DR 2800 spectrophotometer (Hach-Lange GmbH, Düsseldorf, Germany), as described by Alves et al. (2020). Total sulfide species were calculated from the measured dissolved sulfide values, considering the dissociation constants of the acid and pH = 7.5.

## Results

Three different stable glycerol-degrading cultures, coded as Gly-M, Gly-P and Gly-S, were obtained after successive transfers under different environmental conditions, exhibiting distinct microbial composition and product profile (Fig. 1, Tables 1 and 2), producing mainly methane, propionate and acetate, respectively.

**Table 1:** Composition of dominant species in the highly enriched glycerol-degrading cultures

Enrichment culture	Closest relative	Phylum/class^a^	Identity (%)^b^	Coverage (%)
Gly-M	*Solidesulfovibrio alcoholivorans* strain DSM 233	*Thermodesulfobacteriota*/*Desulfovibrionia*	99.8	96
	*Methanofollis liminatans* DSM 4140 strain GKZPZ	*Euryarchaeota*/*Methanomicrobia*	98.8	100
Gly-P	*Propionivibrio pelophilus* strain asp66	*Pseudomonadota*/*Betaproteobacteria*	98.6	99
Gly-S	*Desulfolutivibrio sulfoxidireducens* strain DSM 107105	*Thermodesulfobacteriota/Desulfovibrionia*	98.3	100

^a^Classified using the RDP Classifier

^b^Results of sequence analysis on NCBI BLAST

**Table 2:** Product profile of the glycerol-degrading enrichments

Enrichment culture	Carbon source	Incubation time (*d*)	Main products formed
Gly-M	Glycerol, 10 mmol L^−1^	10	Methane and acetate
Gly-P	Glycerol, 10 mmol L^−1^	12	Propionate
Gly-S	Glycerol, 10 mmol L^−1^ (with sodium sulfate as electron acceptor)	14	Acetate

Methanogenic enrichment series, Gly-M, resulted in a co-culture of a bacterium closely related to *Solidesulfovibrio alcoholivorans* (99.8% 16S rRNA gene identity) and a hydrogenotrophic methanogen closely related to *Methanofollis liminatans* (98.8% 16S rRNA gene identity) (Table 1). Further transfers of the glycerol-degrading culture Gly-M led to a loss of the methane production ability in some of these cultures, and production of propionate was observed (Fig. 1). These enrichments were continued, and a highly specialized fermentative propionate-producing culture composed of a bacterium closely affiliated with the *Propionivibrio pelophilus* asp66 strain (98.6% 16S rRNA gene identity) was obtained (Fig. 1, Tables 1 and 2). This culture was designated *Propionivibrio* sp. strain Gly-P, or simply strain Gly-P or Gly-P culture. Additionally, microscopic observations of the Gly-P culture revealed that it was mainly composed of one morphotype (Fig. S1a), Gram (-) cells, which was in line with the results from the molecular characterization of this culture (Table 1). In the cultures in which sulfate was added to the anaerobic medium (Fig. 1), the microbial specialization resulted in a distinct culture, composed of only one morphotype (Fig. S1b), Gram (-) cells, closely related to *Desulfolutivibrio sulfoxidireducens* strain DSM 107105 (98.3% 16S rRNA gene identity) (Table 1). This culture was named *Desulfolutivibrio* sp. strain Gly-S or simply strain Gly-S or Gly-S culture.

**Fig. 2:**
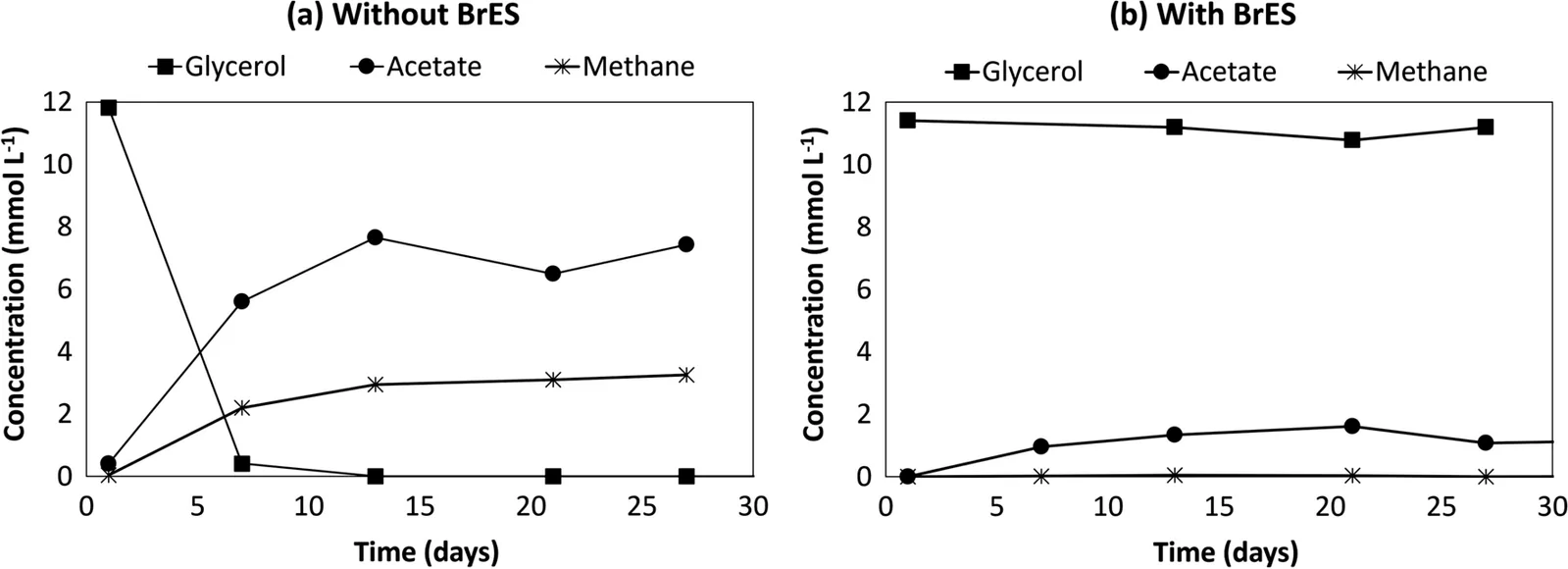
Glycerol conversion by Gly-M culture: (**a**) incubated without BrES or (**b**) incubated with BrES

The Gly-M co-culture was able to use glycerol in approximately 10 days and formed acetate and methane (Table 2, Fig. 2a). When the co-culture Gly-M was incubated with BrES, a specific inhibitor of the methanogens (DiMarco et al. 1990), glycerol was not degraded, as shown in Fig. 2b. Methane was not produced, and only a residual quantity of acetate could be detected (Fig. 2b).

**Fig. 3:**
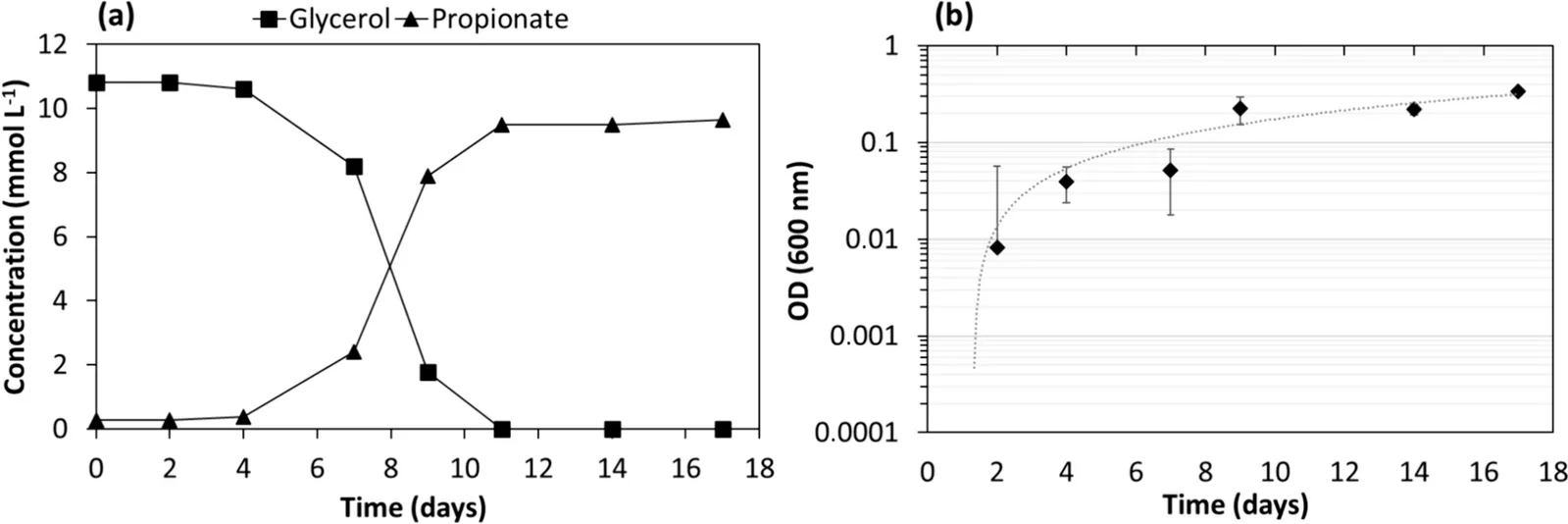
Glycerol conversion by Gly-P culture coupled to propionate production (**a**) and bacterial growth curve on a semi-logarithmic scale (**b**)

*Propionivibrio* sp. strain Gly-P was able to use aspartate (20 mmol L^−1^), with the production of acetate (~ 4 mmol L^−1^) and propionate (~ 9 mmol L^−1^). When grown with glycerol (10 mmol L^−1^), Gly-P produced mainly propionate (9.5 mmol L^−1^) and acetate (0.4 mmol L^−1^) in 12 days of fermentation (Fig. 3, Table 3). These values correspond to a propionate yield of 0.88 mmol propionate per mmol of glycerol consumed (0.71 mg mg^−1^) and a carbon recovery (considering the CO_2_ possibly produced concomitantly with acetate) of 92% (Table 3). It should be mentioned that biomass production was not included in this balance.

**Table 3:** Fermentation profile of *Propionivibrio* sp. strain Gly-P, grown under different initial glycerol concentrations

Initial [glycerol] *theoretical*	Initial [glycerol] *measured*	Glycerol consumed	Products (mmol L^−1^)			Carbon recovery^a^	Prop yield^b^	Ac yield^b^	Succ yield^b^
(mmol L^−1^)	(mmol L^−1^)	(%)	[Prop]	[Ac]	[Succ]	(%)	(mmol mmol^−1^)	(mmol mmol^−1^)	(mmol mmol^−1^)
10	11	100	9.5	0.4	0	92	0.88	0.037	0.000
30	32	100	26.2	1.9	1.7	93	0.82	0.059	0.053
50	42	82	27.1	2.8	2.8	96	0.80	0.082	0.082
100	77	36	22.9	2.4	1.3	97	0.84	0.086	0.047
200	172	15	20.3	2.3	0.8	90	0.78	0.088	0.032

*Prop* propionate, *Ac* acetate, *Succ* succinate.

^a^Carbon in products/carbon in glycerol consumed. It was assumed that one mole of CO_2_ was produced per mole acetate formed, and one mole of CO_2_ was fixed per mole succinate produced. Biomass production was not included in this balance.

^b^Expressed per mole of glycerol consumed.

*Propionivibrio* sp. strain Gly-P was also able to ferment glycerol up to a concentration of approximately 200 mmol L^−1^. However, for glycerol concentrations of 100 mmol L^−1^ and 200 mmol L^−1^, only 36% and 15% of the glycerol added was consumed, respectively, after 12 days of fermentation (Table 3). Nevertheless, propionate yields remained similar for all the concentrations studied, with an average value of 0.82 ± 0.04 mmol mmol^−1^ (0.66 ± 0.03 mg mg^−1^).

Acetate yields increased with the increasing glycerol concentrations tested, accompanied by decreasing propionate/acetate molar ratios (from 23.8 to 8.9 for 10 mmol L^−1^ to 200 mmol L^−1^ glycerol) (Table 3). Succinate yields increased up to a glycerol concentration of 50 mmol L^−1^ and decreased thereafter (Table 3). Nevertheless, succinate and acetate were always produced in relatively low amounts, leading to a propionate purity in the medium between 80 and 97%. Glycerol consumption rate and propionate production rate increased for glycerol concentrations up to 50 mmol L^−1^ but decreased thereafter (data not shown).

**Fig. 4:**
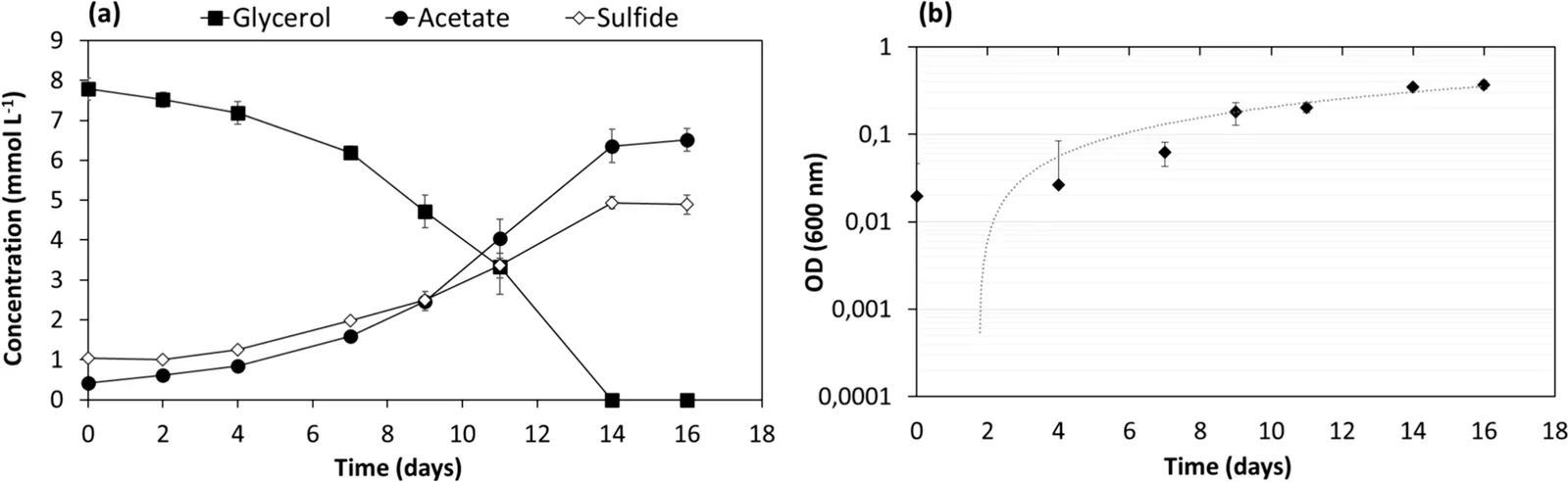
Glycerol conversion by Gly-S culture coupled to acetate production and dissolved sulfide formation (***a***); bacterial growth curve on a semi-logarithmic scale (***b***)

**Fig. 5:**
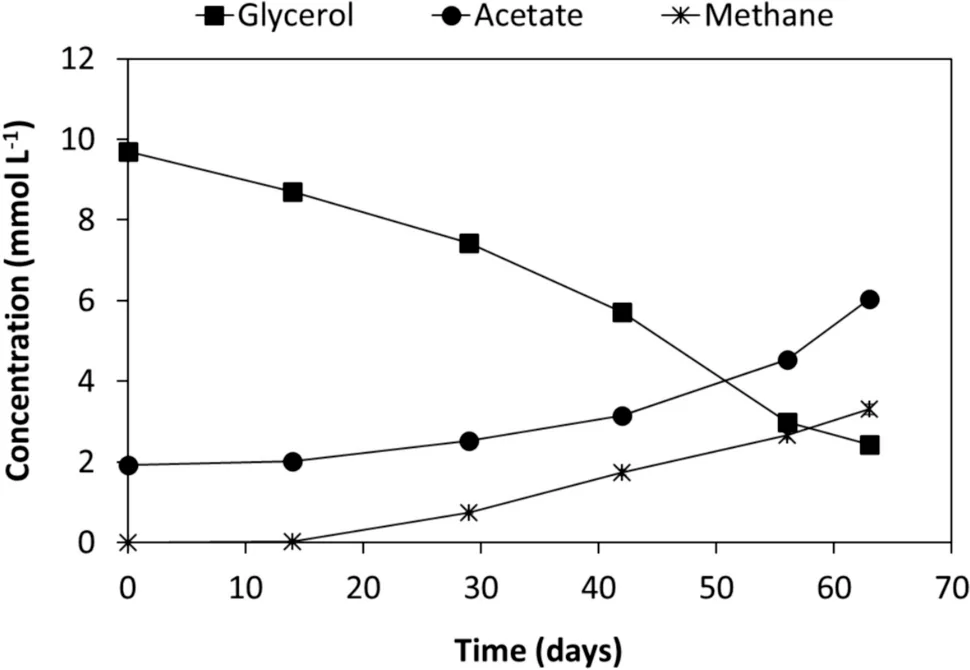
Glycerol conversion and acetate and methane production over time by culture Gly-S incubated with *Methanobacterium formicium*, in the absence of sulfate

*Desulfolutivibrio* sp. strain Gly-S was able to grow with glycerol, by oxidation to acetate coupled to sulfate reduction (Fig. 4, Table 4). When grown with an initial glycerol concentration of 10 mmol L^−1^, glycerol was converted to acetate in 14 days (Table 2, Fig. 4), with an acetate yield (mmol acetate per mmol of glycerol consumed) of 0.84 (Table 4). For higher glycerol concentrations, from 30 to 173 mmol L^−1^, the glycerol consumed by strain Gly-S was practically the same in all the cases, i.e., approximately 23 mmol L^−1^. Acetate (around 14–16 mmol L^−1^) and sulfide (approx. 16 mmol L^−1^) were formed at concentrations that were similar for all the assays (Table 4). *Desulfolutivibrio* sp. strain Gly-S was also able to grow with other alcohols, namely ethanol, propanol and 1-butanol, coupled to sulfate reduction, with acetate as the main product obtained in those situations (data not shown). In the absence of sulfate, no growth was observed, for all the different substrates tested. Glycerol was also degraded by *Desulfolutivibrio* sp. strain Gly-S in the absence of sulfate when incubated with *Methanobacterium formicicum* (Fig. 5).

**Table 4:** Glycerol conversion profile of *Desulfolutivibrio* sp. strain Gly-S, grown under different initial glycerol concentrations, with sulfate as electron acceptor

Initial [glycerol] *theoretical*	Initial [glycerol] *measured*	Glycerol consumed	[Acetate]	Acetate yield^a^	[Sulfide]	Total sulfide	Electron recovery^b^
**(mmol L**^**−1**^**)**	**(mmol L**^**−1**^**)**	**(%)**	**(mmol L**^**−1**^**)**	**(mmol mmol**^**−1**^**)**	**(mmol L**^**−1**^**)**	**(mmol L**^**−1**^**)**	**(%)**
10	8	100.0	6.5	0.84	4.9	5.7	96.8
30	31	85.7	16.4	0.62	13.7	15.7	79.4
50	45	51.4	14.3	0.62	13.6	15.6	89.8
100	85	26.6	14.7	0.65	13.6	15.6	91.5
200	173	13.4	16.4	0.70	14.1	16.1	92.6

The theoretical sulfide value was calculated considering the amount of glycerol degraded and the stoichiometry of the reaction (Eq. 5).

^a^Expressed per mole of glycerol consumed.

^b^Electron recovery = total sulfide formed/theoretical sulfide value.

## Discussion

Although starting from the same inoculum, microbial specialization was evidenced for glycerol conversion, with organisms from three different phyla—*Euryarchaeota*, *Thermodesulfobacteriota* and *Pseudomonadota*—being found in the three distinct glycerol-degrading cultures. The use of anaerobic granular sludge proved to be an efficient microbial platform for the production of biocommodities, such as propionate, methane and/or acetate. The different products generated by the stable cultures suggest metabolic specialization, with glycerol being degraded through different pathways (Fig. S2).

In the Gly-M co-culture, glycerol was converted to acetate and H_2_ (Eq. 2), with subsequent conversion of hydrogen to methane by the hydrogenotrophic methanogen (Eq. 3). The conversion stoichiometry is shown in Eq. 4, and the possible metabolic pathway is illustrated in Fig. S2a. The absence of aceticlastic methanogens allows acetate production, for potential use as commodity chemical. The activity of the hydrogenotrophic methanogen mitigates the thermodynamic constraints associated with high hydrogen partial pressure, contributing to the redox balance by removing the excess reducing power (Fig. S2a).

2 $${ \mathrm{C}}_{3}{\mathrm{H}}_{8}{\mathrm{O}}_{3}+2 {\mathrm{H}}_{2}\mathrm{O}{\to \mathrm{C}}_{2}{\mathrm{H}}_{3}{{\mathrm{O}}_{2}}^{-}+{{\mathrm{HCO}}_{3}}^{-}+3{\text{ H}}_{2}+2{\text{ H}}^{+}$$

3 $$4 {\mathrm{H}}_{2}+{{\mathrm{HCO}}_{3}}^{-}+{\mathrm{H}}^{+}\to {\mathrm{CH}}_{4}+3{\text{ H}}_{2}\text{O }$$

4 $${\mathrm{C}}_{3}{\mathrm{H}}_{8}{\mathrm{O}}_{3}\to {\mathrm{C}}_{2}{\mathrm{H}}_{3}{{\mathrm{O}}_{2}}^{-}+0.75\text{ C}{\mathrm{H}}_{4}+0.25\text{ HC}{{\mathrm{O}}_{3}}^{-}+0.25 {\mathrm{H}}_{2}\mathrm{O}+1.25{\text{ H}}^{+}$$

Syntrophic relationships between fermentative bacteria (e.g., *Thermoanaerobacter* species, *Escherichia coli*) and methanogens were reported to facilitate glycerol fermentation (Magalhães et al. 2020; Richter and Gescher 2014; Zhang et al. 2015). However, co-culture Gly-M incubated with BrES was not able to degrade glycerol, nor to produce methane (Fig. 2b), highlighting that the presence of the methanogen is essential for glycerol conversion, and pointing to the occurrence of an obligatory syntrophic relationship.

Association between sulfate-reducing bacteria, such as *Desulfovibrio* species and methanogens, has been reported in the absence of sulfate, mostly regarding ethanol and lactate degradation (Rabus et al. 2006). Still, *Solidesulfovibrio alcoholivorans*, *S. fructosovorans* and *S. carbinolicus* were reported to degrade glycerol without sulfate in the presence of *Methanospirillum* *hungatei* (Qatibi et al. 1998; Qatibi et al. 1991a, b). From the known *Solidesulfovibrio* strains (former *Desulfovibrio* sp.), only *S. fructosovorans* DSM 3604 (Qatibi et al. 1998) and *S. carbinolicus* strain EDK82 (Nanning and Gottschal 1986) are able to perform glycerol fermentation. All the other strains that are known to degrade glycerol can only do it in the presence of sulfate as an external electron acceptor or in syntrophy with a methanogen.

After several transfers of Gly-M culture, propionate production was observed, leading to a new line of enrichments—Gly-P. Propionate production has attracted significant attention due to its importance as a chemical building block widely used in various industries, including feed and food preservatives, herbicides, cosmetics, plastics and pharmaceuticals (Ahmadi et al. 2017; Gonzalez-Garcia et al. 2017). Glycerol conversion to propionate results in higher production yields and less by-products compared to other substrates (Barbirato et al. 1997; Chen et al. 2016; Coral et al. 2008; Dishisha et al. 2015), mainly due to the high reduction state of glycerol. Moreover, this conversion is redox-neutral (Fig. S2b) and yields more energy (Barbirato et al. 1997).

The closest cultured relative of strain Gly-P, *P. pelophilus* strain asp66, was reported to be not able to degrade glycerol. The same is the case for all the other *Propionivibrio* species described (Brune et al. 2002; Hansen et al. 1990; Tanaka et al. 1990; Thrash et al. 2010). In fact, by analyzing the genome of *P. pelophilus* strain asp66 (DSM 12018^ T^), at the Integrated Microbial Genomes (IMG) (https://img.jgi.doe.gov/) and at The National Center for Biotechnology Information (NCBI) (https://www.ncbi.nlm.nih.gov/) genomic platforms, it was possible to confirm that this bacterium lacks the genes encoding for the enzymes that are directly linked to glycerol utilization, such as glycerol dehydratase, glycerol dehydrogenase or glycerol kinase (Clomburg and Gonzalez 2013). This fact, together with a lower 16S rRNA gene identity than 98.7% (which is the threshold for the same species), points out that strain Gly-P could potentially represent a novel *Propionivibrio* species. Both Gly-P and *Propionivibrio pelophilus* strain asp66 (Hansen et al. 1990), its closest relative, were unable to grow with ethanol, propanol, butanol and succinate.

For all glycerol concentrations tested, propionate yield remained relatively constant (Table 3), corroborating the findings of Chen et al. (2016), who showed a minimal impact on propionate yield with increasing glycerol concentrations. Barbirato et al. (1997) also reported similar or lower propionate yields for *Acidipropionibacterium acidipropionici* (Nouioui et al. 2018), *Cutibacterium acnes* (Nouioui et al. 2018) and *Anaerotignum propionicum* (Ueki et al. 2017). Additionally, Zhang and Yang (2009) reported propionate yields from glycerol of 0.67–0.88 mmol mmol^−1^ (0.54–0.71 g g^−1^) by metabolically engineered *Propionibacterium acidipropionici*.

With the increase in glycerol concentrations, succinate and acetate yields started to increase (Table 3), most probably due to inhibition by propionate accumulation. Succinate is a precursor to propionate (Fig. S2b), and its accumulation in the assays (although at low concentrations) suggests inhibition of the succinate pathway of propionate formation. At the same time, the metabolic flux is also being redirected towards acetate production. End-product inhibition typically constrains propionic acid fermentation processes (Zhang and Yang 2009). Among volatile fatty acids, propionate concentrations can have the most significant inhibitory effect on glycerol degradation (Chen et al. 2016; Zhang et al. 2015), due to product-mediated inhibition on cell growth and metabolic activity (Blanc and Goma 1987).

Regarding to Gly-S culture (Fig. S2c), and considering the stoichiometry of the reaction shown in Eq. 5, electron recovery between 79.4 and 96.8% was calculated for the different glycerol concentrations studied (Table 4).

5 $${\mathrm{C}}_{3}{\mathrm{H}}_{8}{\mathrm{O}}_{3}+0.75\text{ S}{{\mathrm{O}}_{4}}^{2-}\to {\mathrm{C}}_{2}{\mathrm{H}}_{3}{{\mathrm{O}}_{2}}^{-}+{{\mathrm{HCO}}_{3}}^{-}+0.75\text{ H}{\mathrm{S}}^{-}+1.25 {\mathrm{H}}^{+} +{\mathrm{H}}_{2}\mathrm{O}$$

For glycerol concentrations higher than 30 mmol L^−1^, substrate inhibition strongly constrained the activity of this culture (Table 4). The capacity of its closest relative, *Desulfolutivibrio sulfoxidireducens*, to utilize glycerol was previously reported by Bak and Pfennig (1987), although showing very slow glycerol consumption and very poor growth. Chen et al. (2019) compared synthetic communities comprising a sulfate reducer (*D. vulgaris* strain Hildenborough) and two methanogens, assembled as syntrophic co- or tri-cultures. When the cultures were placed with sulfate, the methane production was highly diminished, which was attributed to a metabolic shift in bacteria towards respiration with sulfate, leading to a disruption in the methanogenic population (Chen et al. 2019). A similar biochemical conflict between the different metabolic processes (sulfate reduction and methanogenesis as biological electron acceptor) most probably shaped the microbial specialization observed in Gly-S culture.

When Gly-S culture was incubated without sulfate, no growth occurred, but when co-incubated with a methanogenic partner, such as *Methanobacterium formicicum*, glycerol was slowly converted to acetate and methane, showing that the methanogen was consuming the hydrogen/formate generated from glycerol and working as biological electron acceptor. It is worth recalling that Gly-S culture was enriched from a methanogenic granular sludge, which most probably influenced its metabolic traits.

In summary, this work explores the microbial diversity and metabolic specialization of anaerobic microorganisms involved in glycerol conversion and valorization. Three distinct stable cultures were developed, under different environmental conditions, using microbial mixed cultures as biocatalysts. These cultures have the ability to grow and convert glycerol into biocommodities that can be used in industrial applications or as energy carriers. Syntrophic (Gly-M), fermentative (Gly-P) and sulfate-reducing (Gly-S) cultures were obtained, allowing the sustainable treatment and valorization of glycerol. This work contributes to tackle the bottleneck of biodiesel production, caused by the surplus of glycerol. The specialization of cultures that was observed led to product diversification, also contributing to anaerobic process valorization. It was indicated that the top-down design of the microbiome is a promising strategy for not only utilization of troublesome waste but also suitable for dedicated platform chemical production (Lawson et al. 2019). The investment in biological methods of environmental-friendly nature is a demand for application at an industrial level and the development of novel bio-based technologies.

## Supplementary Information

Below is the link to the electronic supplementary material.

Supplementary file1 (PDF 356 KB)

## Data Availability

*Propionivibrio* sp. strain Gly-P and *Desulfolutivibrio* sp. strain Gly-S were deposited in the China General Microbiological Culture Collection Center (CGMCC) under accession no. CGMCC 1.18109 and 1.18110, respectively. Nucleotide sequencing data have been submitted to the European Nucleotide Archive (ENA) under the study accession no. PRJEB72408. All other relevant data generated and analyzed during this study, which include experimental data, are included in this article and its supplementary information.
