# Supplementary material for: Metabolic versatility of anaerobic sludge towards platform chemical production from waste glycerol
Source: Appl Microbiol Biotechnol. 2024 Jul 16;108(1):419. doi: 10.1007/s00253-024-13248-6 (PMC11252210; doi:10.1007/s00253-024-13248-6)
Supplement: Supplementary file 1 — Supplementary file1 (PDF 356 KB) [file 253_2024_13248_MOESM1_ESM.pdf]

Metabolic versatility of anaerobic sludge towards platform chemicals production from waste glycerol

Carla P Magalhães[1*], Joana I Alves[1,2*], Anna Duber[3], Piotr Oleskowicz-Popiel[3], Alfons JM Stams[1,4], Ana J Cavaleiro[1,2#]

[*] The authors contributed equally to this work

Affiliations:

[1] CEB - Centre of Biological Engineering, University of Minho, Braga, Portugal;

[2] LABBELS—Associate Laboratory, Braga/Guimarães, Portugal;

[3] Water Supply and Bioeconomy Division, Faculty of Environmental Engineering and Energy, Poznan University of Technology, Poznan, Poland;

[4] Laboratory of Microbiology, Wageningen University & Research, Wageningen, The Netherlands

[#] **Corresponding Author:** Ana Júlia Cavaleiro, acavaleiro@ceb.uminho.pt

Supplementary Information

Table S1 Summary of the ecophysiological, biochemical and molecular characterization of Gly-M, Gly-P and Gly-S enriched cultures

	**Gly-M**	**Gly-P**	**Gly-S**
**Growth media:** bicarbonate-buffered mineral salt medium	+	+	+
**Growth under the control condition:** glycerol 10 mmol L^{-1}	+	+	+
**Use of sodium sulfide as electron acceptor** (20 mmol L^{-1})	-	-	+
**Growth with** and **without BrES addition** (20 mmol L^{-1})	+	-	-
**Potential syntrophic growth** with a methanogenic partner	-	-	+
**Growth in the presence of different glycerol concentrations:** 10, 30, 50, 100 and 200 mmol L^{-1} .	-	+	+
**Growth with ethanol, propanol or 1-butanol** (10 mmol L^{-1})**, aspartate** (20 mmol L^{-1}) **or succinate** (20 mmol L^{-1})	-	+	-
**Growth with ethanol, propanol and 1-butanol** at different concentrations (10, 20, 30 and 40 mmol L^{-1})	-	-	+
**DNA extraction + 16S rRNA gene sequence** (Sanger)	+	+	+
**Gram staining** and **microscopic observation**	-	+	+
**Purity check:** yeast extract (2 g L^{-1}); glucose (10 mmol L^{-1}); or pyruvate (10 mmol L^{-1})	-	+	+

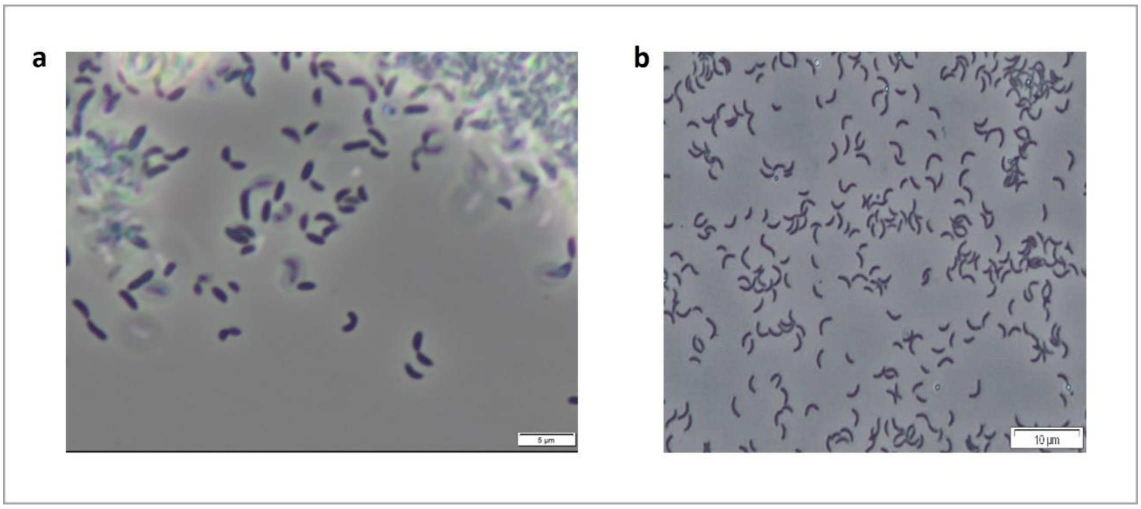

Fig. S1 Phase-contrast micrographs of pure cultures growing in glycerol showing the predominance of one unique morphotype for each culture: **a** Gly-P culture. Bar, 5 μm; **b** Gly-S culture. Bar, 10 μm

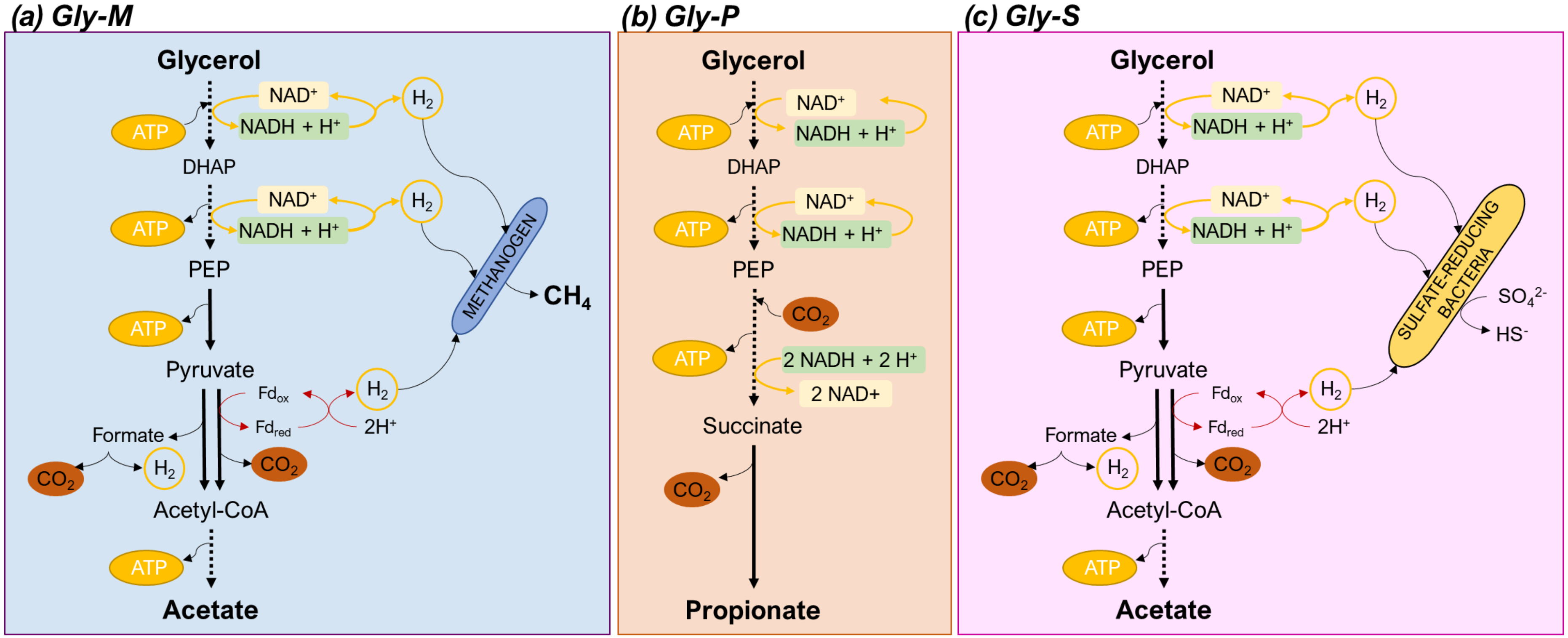

Fig. S2 Possible metabolic pathways for glycerol conversion the enriched cultures: **a** Gly-M, **b** Gly-P and **c** Gly-S. Broken lines mean multiple steps. Abbreviations: DHAP, dihydroxyacetone phosphate; PEP, phosphoenolpyruvate; Acetyl-CoA, acetyl-coenzyme A; Fd_{ox}, oxidized ferredoxin; Fd_{red}, reduced ferredoxin (adapted from Viana et al., 2012; Clomburg and Gonzalez, 2013; Magalhães et al., 2020).
